# Exceptional Response to Trastuzumab Deruxtecan in a Patient With Recurrent Ovarian Clear Cell Carcinoma With Human Epidermal Growth Factor Receptor 2 Expression

**DOI:** 10.1200/PO.23.00686

**Published:** 2024-06-21

**Authors:** Ben L. Kong, Jayne M. Stommel, Jamie M. Keck, David Kilburn, Aaron Streblow, Julian Egger, Allison L. Creason, Christopher G. Suciu, Alexander R. Guimaraes, Christopher L. Corless, Gordon B. Mills, Tanja B. Pejovic

**Affiliations:** ^1^ Knight Cancer Institute, Oregon Health & Science University, Portland, OR; ^2^ Division of Oncological Sciences Knight Cancer Institute, Oregon Health and Science University, Portland, OR; ^3^ Oregon Health & Science University School of Medicine, Portland, OR; ^4^ Department of Pathology, Oregon Health & Science University, Portland, OR; ^5^ Department of Diagnostic Radiology, Oregon Health & Science University, Portland, OR

## Abstract

Case report of a HER2-expressed ovarian clear cell carcinoma with exceptional response to trastuzumab deruxtecan.

## Introduction

Ovarian clear cell carcinoma (OCCC) represents approximately 10% of epithelial ovarian carcinomas and has distinct characteristics and molecular profiles. OCCC is generally intrinsically chemotherapy-resistant and has a poor prognosis.^1,2^ Because of the relative rarity of the disease, enrollment in clinical trials is often difficult, creating uncertainty in determining optimal treatment approaches.

Human epidermal growth factor receptor 2 (HER2) has been extensively studied in breast cancer and has both prognostic and therapeutic roles. However, the role of HER2 in ovarian cancer is less clear.^3^ HER2 is more frequently expressed in OCCC (43%) than in other ovarian cancer subtypes such as serous (21%), ovarian endometrioid (23%), and mucinous (30%).^4^ When OCCC cell lines and xenograft models were treated with trastuzumab, it exhibited dose-dependent inhibition that correlates with HER2 expression.^4^

A Phase II trial of trastuzumab for recurrent/refractory ovarian or primary peritoneal cancer with HER2 overexpression (on the basis of immunohistochemistry [IHC] 2+ or 3+) led to an overall objective response rate (ORR) of 7.3%. Most of the tumors were serous (65.9%); only seven cases were clear cell.^5^ Although trastuzumab alone may not be effective, there is interest in fam-trastuzumab deruxtecan-nxki (Enhertu, T-DXd, DS-8201a), which is US Food and Drug Administration–approved for metastatic breast cancer, non–small cell lung cancer, and gastric or gastroesophageal junction adenocarcinoma.^6^ This compound is an antibody-drug conjugate (ADC) consisting of an anti-HER2 antibody linked to a topoisomerase I inhibitor, and designed to deliver a potent cytotoxic payload to HER2-expressing tumor cells. Furthermore, a bystander effect has been described whereby the payload targets neighboring cells, potentially increasing efficacy and/or toxicity.^7^

Here, we describe an exceptional response to trastuzumab deruxtecan in a HER2-expressing, advanced, recurrent OCCC that was treated in the Molecular Mechanisms of Tumor Evolution and Resistance to Therapy (MMTERT) observational study, which aims to identify actionable alterations in serial tumor samples using multiomic analytics.^8^ Written informed consent was obtained from the patient for the publication of this article.

## Case Presentation

A 67-year-old Caucasian woman (she/her) presented to her primary care provider in 2021 with abdominal swelling and bloating. Paracentesis removed 3 L of ascitic fluid and cytology demonstrated adenocarcinoma of unknown origin. The patient has a history of endometriosis and previously undergone total abdominal hysterectomy and bilateral salpingo-oophorectomy in 2020 for symptomatic, benign fibroids with elevated cancer antigen (CA)-125 (reported by the patient to be in the 300s). The oncologist and the tumor board reviewed the original pathology and agreed with the assessment.

With suspicion of a gynecologic origin, a diagnostic laparoscopy was performed and pathologic findings were consistent with Stage IIIC clear cell carcinoma of Mullerian origin. Molecular analysis performed through an in-house somatic next-generation sequencing (NGS) panel of 225 genes (GeneTrails Comprehensive Solid Tumor Panel) and a send-out germline NGS panel of 47 genes (Invitae Common Hereditary Cancers Panel) was negative for genetic aberrations.

Because of the extent of disease, the patient was not a surgical candidate and was initiated on neoadjuvant chemotherapy (carboplatin [AUC 6 intravenous infusion once every 3 weeks]/paclitaxel [175 mg/m^2^ intravenous infusion once every 3 weeks]). With minimal response and elevating CA-125, an exploratory laparotomy was successfully performed to R0 (no residual disease) and omentum tissue was sent for analysis.

**FIG 1.**
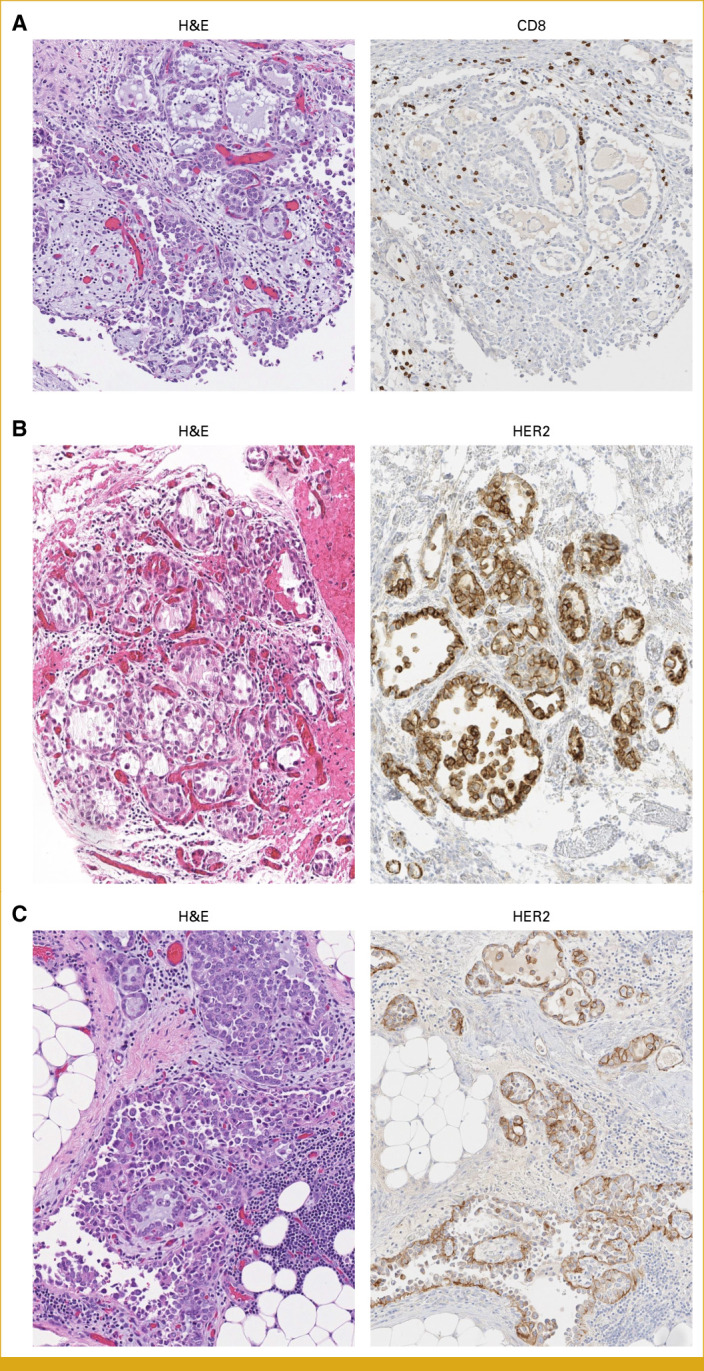
(A) H&E and CD8 immunohistochemistry on the exploratory laparotomy specimen. Original magnifications 10×. (B) H&E and HER2 immunohistochemistry on the exploratory laparotomy specimen. Original magnifications 12×. (C) H&E and HER2 immunohistochemistry on the diagnostic laparoscopy specimen. Original magnifications 10×. HER2, human epidermal growth factor receptor 2.

For maintenance, pembrolizumab (200 mg intravenous infusion once every 3 weeks) was started on the basis of marked peritumoral CD4 and CD8 cell infiltration (Fig 1A) and previous low positive PD-L1 (combined positive score = 2). After Cycle 1, there was rapid enlargement and new abdominal lymph nodes suggesting true progression, and bevacizumab (15 mg/kg intravenous infusion once every 3 weeks) was added in Cycle 2. The CA-125 continued to rise, and cyclophosphamide (50 mg by mouth once daily) was added to form a triplet regimen to potentially enhance T-cell infiltration and decrease local immunosuppression for recurrent, platinum-resistant disease.^9^ It was discontinued after a week because of severe mouth sores. Resuming pembrolizumab/bevacizumab, there was minimal decrease in lymphadenopathy and new splenic lesions.

At this time, the MMTERT multidisciplinary molecular tumor board reviewed the multiomics results and suggested lenvatinib/pembrolizumab (if possibility of endometrial cancer with history of endometriosis), clinical trials with *ATM* loss criteria, or T-DXd on the basis of strong HER2 expression (Fig 1B). The lenvatinib (4 mg by mouth once daily)-based combination was started thereafter, but eventually discontinued as it became intolerable (stomatitis, drowsiness, and fatigue) without any perceived benefit.

**FIG 2.**
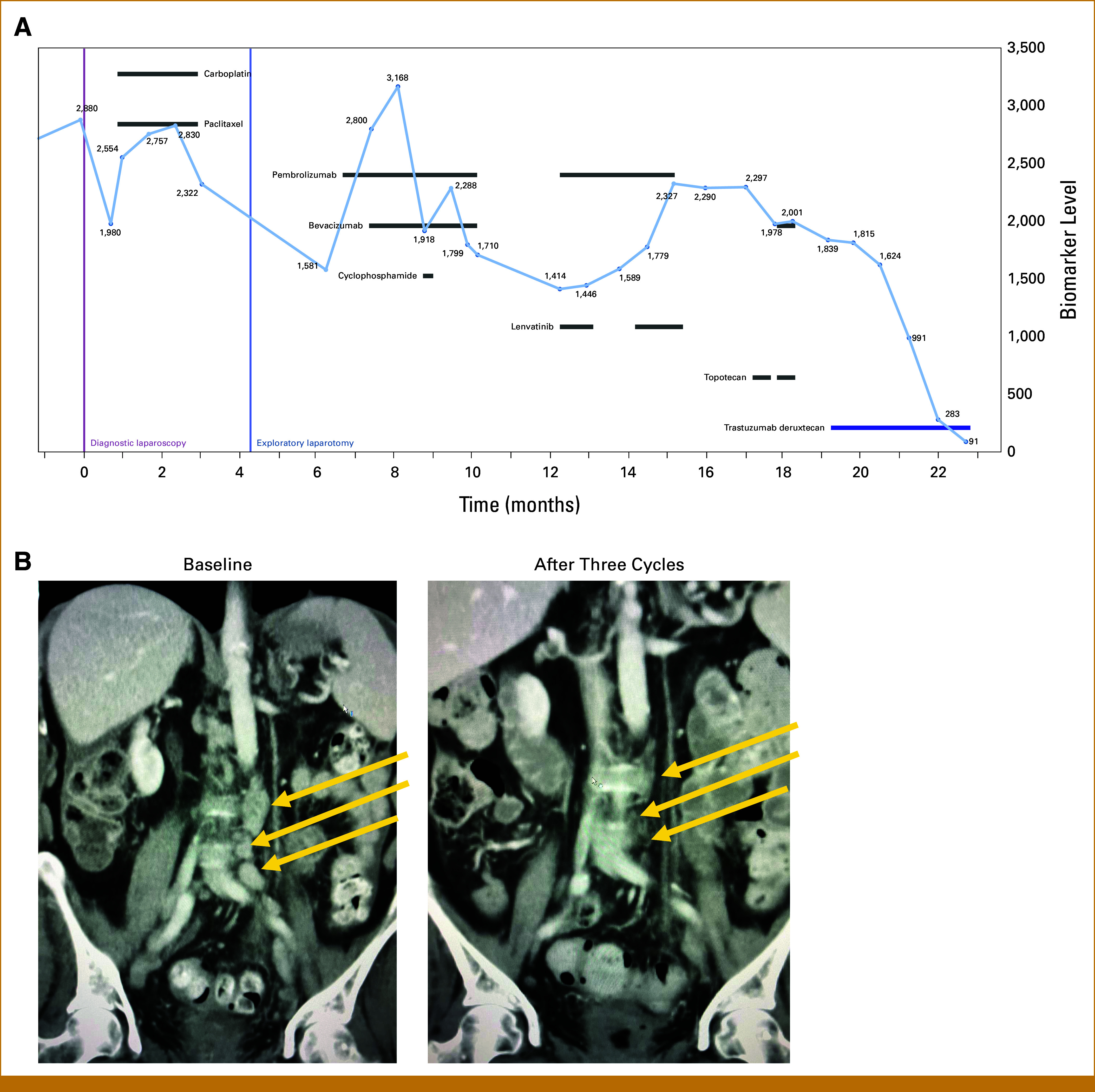
(A) Overview of clinical course and treatment in conjunction with serum tumor marker (CA-125). After the initiation of trastuzumab deruxtecan (5.4 mg/kg intravenous infusion once every 3 weeks), there was imaging confirmed partial response along with a significant decrease in CA-125. (B) Coronal CT images at baseline showed extensive retroperitoneal adenopathy (arrows) in the left periaortic space. After three cycles of treatment with trastuzumab deruxtecan, there was amelioration and normalization of adenopathy with normal sized lymph nodes. CA, cancer antigen; CT, computed tomography.

With limited options, topotecan (2 mg/m^2^ intravenous infusion once every 2 weeks)/bevacizumab (10 mg/kg intravenous infusion once every 2 weeks) was selected as a bridging therapy while the drug acquisition process was initiated for T-DXd (5.4 mg/kg intravenous infusion once every 3 weeks). A restaging computed tomography scan after three cycles of T-DXd showed a significant decrease in the retroperitoneal and pelvic nodes, splenic lesions, ascites, and pleural effusion consistent with a partial response measured by RECIST 1.1 in addition to an approximately 50% decrease in CA-125 (Figs 2A and 2B). Adverse events evaluated with Common Terminology Criteria for Adverse Events v5.0 were manageable and consisted of nausea/vomiting (Grade 1) and fatigue (Grade 2). An on-treatment biopsy was considered to evaluate response biomarkers but was not feasible because of the marked clinical response. After six cycles, there was further response and the patient requested a drug holiday because of personal choice. In 12 weeks, she developed bilateral pleural effusions with an increased size of a pelvic lymph node and has since resumed T-DXd.

## Correlative Results

Testing was performed in the OHSU Knight Diagnostic Laboratories. See Table 1 for a summary of results generated from the omental specimens obtained during the diagnostic laparoscopy procedure and subsequent exploratory laparotomy with tumor debulking (30% tumor content in each).

**TABLE 1.** Comparison of Correlative Results Between the Diagnostic Laparoscopy and Exploratory Laparotomy

Procedure	Diagnostic Laparoscopy	Exploratory Laparotomy
Biopsy location, tumor content	Omentum, 30%	Omentum, 30%
Genomic variants	No somatic aberrations detected No germline aberrations detected	TMB <1 mutations/Mb Microsatellite stable **ATM loss (0.1 copies)** **SMAD4 loss (0.5 copies)** **PMS2 loss (0 copies, VUS)** No ERBB2 amplification detected
RNA transcriptome	No gene fusions detected	No gene fusions detected
Protein (IHC)^a^	**Ki-67 20%-30%** CD4 moderate peritumoral infiltrate CD8 marked peritumoral infiltrate **PD-L1 (22C3 antibody)–positive (TPS = 0; CPS = 2)** HER2-positive (3+)	**Ki-67 50%-60%** CD4 marked peritumoral infiltrate CD8 marked peritumoral infiltrate **PD-L1 (SP263 antibody)–negative (TPS = 0; CPS = 0)** HER2-positive (3+)

NOTE. Bold entries indicate a difference in results.

Abbreviations: CPS, combined positive score; HER2, human epidermal growth factor receptor 2; IHC, immunohistochemistry; Mb, megabase; TPS, tumor proportion score; VUS, variant of unknown significance.

^a^ Ventana 4B5 antibody was used to assess HER2 IHC.

Ovarian clear cell carcinomas maintain both luminal and basolateral membrane features; therefore, HER2 immunohistochemical staining was scored using the guidelines for gastric cancer, as published by the College of American Pathologists.^10^

Multiplex analysis of 67 cancer-associated proteins and phosphoproteins was performed using the Nanostring GeoMx Digital Spatial Profiling (DSP) system. This assay consists of a combination of commercially available antibody modules and a panel of antibodies that were custom-tagged. The assay readout was performed on a Nanostring MAX nCounter system. Because ovarian clear cell carcinoma is relatively rare, the signals from the patient's specimens were compared with a reference cohort of 19 breast carcinomas (a mixture of hormone receptor–positive/HER2-negative, HER2-positive, and triple-negative). Signals for each antibody were reported by quartile relative to the reference cohort. A tissue microarray of well-characterized cancer cell lines was included in each run as a control for antibody performance and for data normalization.

### Exploratory Laparotomy Specimen

DSP detected high expression of total HER2 and phospho-HER2 (Data Supplement, Fig S1). Markers of the PI3 kinase/AKT and MAP kinase pathways were low to average in comparison with the breast cancer reference cohort. To confirm the DSP results, HER2 IHC was performed and found to be 3+ (Ventana 4B5 antibody). Furthermore, immunofluorescence staining confirmed the positive HER2 expression. HER2 staining was both cytoplasmic and nuclear, and was strong in E-cadherin–positive epithelial cells (Data Supplement, Fig S2).

Whole transcriptomic sequencing (Illumina TruSeq RNA exome) did not detect any gene fusions. Compared with The Cancer Genome Atlas ovarian cohort, HER2 RNA expression and HER2 activity were average (Data Supplement, Fig S3).

### Diagnostic Laparoscopy Specimen

On the basis of the striking clinical response to T-DXd, we retrospectively performed IHC on the diagnostic specimen and HER2 was also found to be 3+ (Fig 1C).

## Discussion

Advanced ovarian clear cell carcinoma has a poor prognosis with limited treatment options. Therefore, there is a need to further understand biologic mechanisms to develop effective treatment strategies. Although it has been shown that ovarian cancer can overexpress HER2, minimal clinical benefit with anti-HER2 therapies has been observed in clinical trials; however, the available data derive mostly from serous carcinoma.^5^ Through a multiomics platform, we detected and confirmed strong HER2 expression in OCCC. Biopsy specimens had high HER2 protein expression by IHC and DSP in the absence of gene amplification or increased RNA. Unlike breast cancer, where there are high rates of concordance between HER2 expression and amplification, there is less correlation in ovarian cancer, which may be due to a number of factors such as nonstandardized HER2 testing methods, disease subtypes, or tumoral heterogeneity.^11-13^ Intriguingly, the retrospective analysis of the initial diagnostic specimen revealed strong HER2 IHC, suggesting an opportunity for earlier treatment with a HER2 ADC if HER2 testing had been performed.

The recent development and approval of HER2 ADCs warrants reinvestigating HER2 expression as a therapeutic biomarker in OCCC. DESTINY-PanTumor02 is an ongoing Phase II clinical trial studying T-DXd in solid tumors with HER2 expression (2+ or 3+ by IHC; ClinicalTrials.gov identifier: NCT04482309). Preliminary results are promising across all tumor types and correlate with HER2 expression with an ORR of 37.1% for all participants and 45% in the ovarian cohort (abstract LBA3000, abstract LBA34).^14,15^ It is unknown if clear cell carcinoma was represented in this trial as the ovarian cancer subtypes were not reported.

It is interesting to note that although both topotecan and deruxtecan are topoisomerase I inhibitors, it remains unclear if the former would have yielded a similar clinical benefit. There were early signs of response with topotecan on the basis of decreasing CA-125, but the change was less compared with T-DXd (9.3% *v* 55% reduction), perhaps owing to the latter's unique delivery mechanism and the bystander effect.

To the best of our knowledge, this is the first report of a major response in a patient with HER2-positive ovarian clear cell carcinoma treated with trastuzumab deruxtecan. The exceptional clinical response we observed suggests that this agent deserves further study in this subtype of ovarian cancer.
